# Assessing treatment response after intravesical bacillus Calmette–Guerin induction cycle: are routine bladder biopsies necessary?

**DOI:** 10.1007/s00345-021-03690-w

**Published:** 2021-04-08

**Authors:** Beppe Calò, Francesca Sanguedolce, Ugo G. Falagario, Marco Chirico, Francesca Fortunato, Emanuel Carvalho-Diaz, Gian Maria Busetto, Carlo Bettocchi, Giuseppe Carrieri, Luigi Cormio

**Affiliations:** 1 Department of Urology and Renal Transplantation, University of Foggia—Ospedali Riuniti of Foggia, Foggia, Italy; 2 Department of Urology, Bonomo Teaching Hospital, Andria (BAT), Italy; 3 Department of Pathology, University of Foggia, Foggia, Italy; 4 Department of Epidemiology, University of Foggia, Foggia, Italy; 5 Department of CUF Urology and Service of Urology, Hospital of Braga, Braga, Portugal

**Keywords:** High-risk Bladder Cancer, BCG response, Bladder biopsy, NMIBC treatment

## Abstract

**Purpose:**

To determine the need for routine bladder biopsies (BBs) in assessing response to the induction cycle of intravesical bacillus Calmette–Guérin (BCG) for high-risk non-muscle-invasive bladder cancer (NMIBC).

**Methods:**

Our prospectively maintained NMIBC database was queried to identify patients with high-risk disease (carcinoma in situ, high-grade Ta/T1) who underwent BBs after BCG induction cycle. Urine cytology, cystoscopy, and BBs findings were evaluated.

**Results:**

A total of 219 patients met the inclusion criteria. Urine cytology was positive in 20 patients and negative in 199; cystoscopy was positive in 35 patients, suspicious in 32 and normal in 152 patients. BBs yielded bladder cancer (BCa) in 43 (19.6%) patients, with a BCa rate of 9.3% in patients with negative cytology and cystoscopy as opposed to 38.0% in patients whereby one or both exams were suspicious/positive. The diagnostic accuracy of urine cytology, cystoscopy, and combined tests was 0.56, 0.70, and 0.71, respectively. The negative predictive value of combined tests was 90.7%. Performing BBs only in patients with positive cytology and/or positive/suspicious cystoscopy would have spared 140 (64%) patients to undergo this procedure while missing BCa in 13 (9.3%) of them, representing 30% of all BCa cases.

**Conclusion:**

Performing BBs only in patients with positive cytology and suspicious/positive cystoscopy would spare 64% of un-necessary BBs but miss a non-negligible number of BCas. While no data are available regarding the potential consequences of missing such BCas, such information should be taken into account in patient’s counselling.

## Introduction

Bladder cancer (BCa) has the ninth incidence rate among all malignancies worldwide, ranking seventh in men and seventeenth in women [1, 2]. In Europe, mortality rates have shown a substantial reduction of ~ 16% in men and ~ 12% in women over the last decade [3], thus suggesting some improvement in disease management.

The first step in treating such tumor is trans-urethral resection of the bladder (TURBT) which allows disease staging and grading. As much as 75% of cases present as non-muscle-invasive disease (NMIBC), which includes Cis, Ta, and T1 pathologically staged tumors [4]. Several clinical and pathological features allow to predict the ability of NMIBC to recur and progress, thus to classify them into low-, intermediate-, and high-risk disease [4].

According to current European Association of Urology (EAU) guidelines, the standard of care for high-risk NMIBC is, after TURBT, intravesical instillations of bacillus Calmette–Guérin (BCG) [4]. The recommended treatment schedule involves an induction course of 6 weekly instillations possibly followed by maintenance of 1–3 years [4]. Open questions remain on how the efficacy of the BCG induction cycle should be assessed; this is a major clinical issue, because, in high-risk NMIBC, prompt detection of recurrence and progression is crucial, while a delay in diagnosis and treatment may be life-threatening [4].

Current EAU guidelines highlight the importance of first cystoscopy at 3 months from TURBT, while the role of bladder biopsies (BBs) in such cases remains a matter of debate. Indeed, the guidelines provide a weak recommendation for patients with high-risk disease having cystoscopy and cytology at 3 months after TURBT and a strong recommendation for such patients having BBs in case of positive cytology and/or positive/suspicious cystoscopy. Available literature provides conflicting data, since some studies suggest that cystoscopy and urinary cytology may be sufficient to assess the efficacy of BCG induction course, thus reserving BBs only to cases whereby such exams yield suspicious/positive findings [5, 6], while others recommend routine BBs [7–9].

The present study, therefore, aimed to determine the diagnostic accuracy of pathway 1, BBs only in case of positive cytology and/or positive/suspicious cystoscopy, as compared to pathway 2, namely routine BBs in assessing efficacy of BCG induction cycle in patients with high-risk NMIBC.

## Patients and methods

Our prospectively maintained NMIBC databases were queried to identify a cohort of patients complying with all the following inclusion criteria: 1) pathological diagnosis of high-risk NMIBC, including Cis, high-grade (HG) Ta, and HG T1 BCa with or without concomitant Cis; 2) having completed the BCG induction course; 3) having undergone urinary cytology and BBs, obviously including cystoscopy, to assess the efficacy of BCG induction cycle; 4) having complete clinical data. Patients lacking one of these data were excluded.

Urinary cytology was taken 3 weeks after the last BCG instillation. BBs were planned 4–6 weeks after having completed the BCG induction course. The procedure was carried out under spinal anaesthesia using white light instruments. Any visible tumor or suspected area was biopsied and/or resected based on its features and surgeon’s judgement; independently, cold cut biopsies were taken from trigon, right and left wall, dome, fundus, and prostatic urethra in males in all cases. Findings of the cystoscopy carried out before BBs were classified as normal (lack of mucosal erythema, sessile, or papillary tumours), suspicious (presence of mucosal erythema, in absence of raised sessile or papillary tumours), and positive (presence of sessile or papillary tumours). All procedures were performed by or supervised by a senior uro-oncologist surgeon.

Two senior pathologists unaware of clinical data reviewed all specimens in accordance with the latest WHO Classification of tumours of the Urinary System and Male Genital Organs [10] and the 2017 TNM staging system [11]. Urine cytology was classified as negative (normal or low-grade) or positive (high-grade) according to the Bladder Consensus Conference Committee of 1998 [12].

The study protocol was approved by the Internal Review Board and carried out in agreement with the Helsinki Declaration recommendations.

### Statistical analysis

The primary study objective was assessment of specificity, sensitivity, positive predictive value (PPV), negative predictive value (NPV), and accuracy of the two clinical policies, namely urinary cytology + cystoscopy *vs.* urinary cytology + routine BBs in detecting BCa after BCG induction cycle. For the purpose of the analysis, suspicious cystoscopy was considered positive.

Categorical variables were compared by the Chi-square or Fisher’s exact test as appropriate, while continuous variables were reported as median and interquartile range and compared by the Mann–Whitney *U* test. Diagnostic accuracy was tested by ROC curve analysis and Youden index.

Finally, we evaluated recurrence and progression rates of patients who underwent RE-TURBT with those who did not, and we compared oncological outcomes according to cytology and cystoscopy results using the Kaplan–Meier method and log-rank test.

Statistical analyses were performed using Stata-SE 15 (StataCorp LP, College Station, TX, USA). All tests were two-sided with a significance level set at *p* < 0.05.

## Results

A total of 219 patients met the inclusion criteria (Table 1). There were 186 men (84.9%) and 33 women (15.1%); their median age was 69.0 years (IQR range 62.0–76.0). Indication for BCG treatment was CIis in 12 cases (5.5%), HG T1 in 144 (65.7%), and HG Ta in 63 (28.7%). Concomitant Cis was also found in 10 cases (4.9%), 3 with Ta and 7 with T1 disease. One-hundred and forty-two (64.8%) patients underwent second-TUR before the BCG induction cycle showing NMIBC in 74 (52.1%) patients, namely Cis in 25 (17.6%), HG Ta in 18 (14.5%), low-grade (LG) Ta in 16 (11.2%), HG T1 in 15 (10.5%) patients, and T0 in 68 (47.9%).

**Table 1:** Patients’ characteristics according to BBs results

	Overall	Negative BBs	Positive BBs	*p* value
Patients, *n* (%)	219	176 (80.4)	43 (19.6)	
Age, yr (IQR)	69.0 (62.0, 76.0)	69.0 (62.0, 76.0)	71.0 (62.0, 76.0)	0.6
Gender, *n* (%)				0.5
Female	33 (15.1)	28 (15.9)	5 (11.6)	
Male	186 (84.9)	148 (84.1)	38 (88.4)	
BCa stage and grade				
Ta HG, *n* (%)	63 (28.7)	55 (87.3)	8 (12.7)	
T1 HG, *n* (%)	144 (65.7)	113 (78.5)	31 (21.5)	
Cis, *n* (%)	12 (5.5)	8 (66.6)	4 (33.4)	
Primary, *n* (%)				0.4
Yes	161 (73.5)	127 (72.2)	34 (79.1)	
No	58 (26.5)	49 (27.8)	9 (20.9)	
Multifocal, *n* (%)				0.6
Yes	129 (58.9)	102 (58.6)	27 (62.8)	
No	90 (41.1)	74 (42.0)	16 (37.2)	
Concomitant Cis, *n* (%)				0.4
Present	10 (4.6)	9 (5.1)	1 (2.3)	
Absent	209 (95.4)	167 (94.9)	43 (97.7)	
Diameter, *n* (%)				**0.021**
> 30 mm	157 (71.7)	133 (75.6)	24 (55.8)	
< 30 mm	62 (28.3)	43 (24.4)	19 (44.2)	
Re-TUR, *n* (%)				0.14
Yes	142 (64.8)	110 (62.5)	32 (74.4)	
No	77 (35.2)	66 (37.5)	11 (25.6)	
Cystoscopy,				
Normal, *n* (%)	152 (69.4)	136 (89.5)	16 (10.5)	** < 0.0001**
Suspicious, *n* (%)	32 (14.6)	27 (84.4)	5 (15.6)	
Positive, *n* (%)	35 (16.0)	13 (37.2)	22 (62.8)	
Urine cytology, *n* (%)				
Negative, *n* (%)	199 (90.9)	164 (82.4)	35 (17.6)	**0.016**
Positive, *n* (%)	20 (9.1)	12 (60.0)	8 (40.0)	

*IQR* Interquartile range; *BCa* Bladder cancer; *HG* High-grade; *Cis* Carcinoma in situ; *Re-TUR* Second transurethral resection

In bold statistically significant values

Table 1 shows patients’ characteristics according to BBs results. Urinary cytology before BBs was positive in 20 (9.1%) patients. Cystoscopy at the time of BBs was suspicious/positive in 67 (30.6%) patients. BBs were positive in 43 (19.6%) patients; specifically, in 8 (40.0%) of the 20 patients with positive cytology and 35 (17.6%) of the 199 negative cytology, in 22 (62.8%) of the 35 patients with positive cystoscopy, 5 (15.6%) of the 32 with suspicious cystoscopy, and 16 (10.5%) of the 152 with normal cystoscopy.

Pathology findings at BBs according to cytology and cystoscopy results are shown in Table 2. Pathway 1 (performing BBs only in patients with positive cytology and/or positive/suspicious cystoscopy) would have spared 63.9% (140/219) of BBs, but would have miss tumor in 9.3% (13/140) of patients, which represent approximately 30% of all BCas, with the majority of missed cancers being Cis. On the other hand, Pathway 2 (routine BBs) avoided the 59 (26.9%) patients with negative cytology and suspicious/positive cystoscopy to undergo unnecessary outpatient cystoscopy.

**Table 2:** Diagnostic yield of combined urinary cytology and cystoscopy as assessed by BBS. Pathway 1: BBs only in case of positive cytology and/or positive/suspicious cystoscopy. Pathway 2, routine BBs

Cytology–Cystoscopy results	# of Patients 219	Positive BBs 43 (19.6%)	Pathology findings
Negative–Negative, *n* (%)	140	13 (9.3%)	HG T1 = 1; HG Ta = 1; Cis = 7; LG Ta = 4
Negative–Positive*, *n* (%)	59	22 (37.3%)	HG T1 = 3; HG Ta = 6; Cis = 4; LG Ta = 8; VH† = 1
Positive–Negative, *n* (%)	12	3 (25.0%)	HG T1 = 1; HG Ta = 1; Cis = 1
Positive–Positive*, *n* (%)	8	5 (62.5%)	HG T2 = 2; HG T1 = 1; HG Ta = 2
Pathway 1	79	30 (38.0%)	HG T1 = 5; HG Ta = 9; Cis = 5; LG Ta = 8; VH† = 1
Pathway 2	219	43 (19.6%)	HG T1 = 6; HG Ta = 10; Cis = 12; LG Ta = 12; VH† = 1

*Includes suspicious findings; †Variant histology

The diagnostic accuracy of urinary cytology, cystoscopy, and the combination of the two diagnostic methods is summarized in Table 3.

**Table 3:** Diagnostic accuracy of urinary cytology, cystoscopy, and of their combination

	Sensitivity	Specificity	PPV	NPV	J	Accuracy
Urine cytology, % (95% CI)	18.6 (8.39, 33.4)	93.2 (88.4, 96.4)	40.0 (22.5, 60.5)	82.4 (80.2, 84.5)	0.12	0.56
Cystoscopy, % (95% CI)	62.8 (46.7, 77.0)	77.3 (70.4, 83.2)	40.3 (32.1, 49.1)	89.5 (85.1, 92.7)	0.40	0.70
Combined, % (95% CI)	69.8 (53.9, 82.8)	72.2 (64.9, 78.6)	38.0 (31.0, 45.5)	90.7 (86.0, 94.0)	0.42	0.71

*PPV* Positive predictive value; *NPV* Negative predictive value; *J* Youden index

Median postoperative hospital stay was 2 days (range 1–3). Three patients suffered temporary urinary retention and one hematuria; all cases were solved by a few days of urethral catheterization. There was no case of hospital re-admission.

**Fig. 1:**
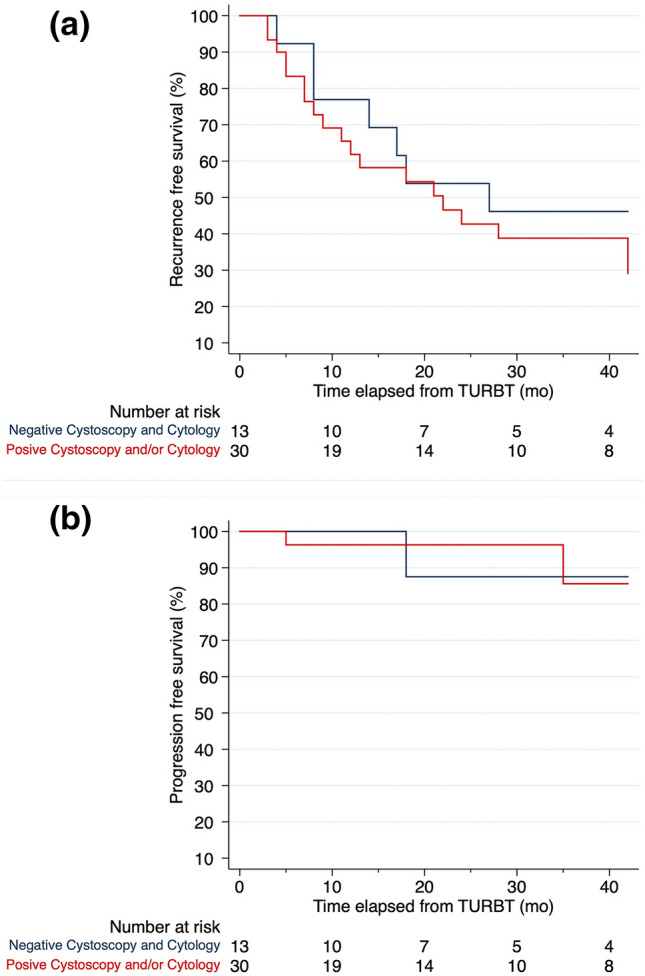
Kaplan–Meier curves showing recurrence (**a**) and progression (**b**) -free survival of patients with a positive bladder biopsy

Median follow-up was 38.0 (IQR: 23.0, 76.0) months. No difference was found in recurrence (25.3% vs 18.2%, *p* 0.227) and progression (8.5% vs 11.7%, *p* 0.513) rates, respectively, in patients who underwent RE-TURBT and in patients who did not. Similarly, within the 43 patients who had a positive bladder biopsy, recurrence and progression rates were similar between 13 patients with negative cytology and cystoscopy and 30 patients who had either one or both the tests positive (recurrence 53.9% vs 63.3%, *p* 0.559; progression 7.7% vs 13.3%, *p* 0.596) with similar estimated recurrence and progression-free survival using the Kaplan–Meier Methods (Fig. 1, *p* = 0.422 and 0.964, respectively).

## Discussion

BCG therapy is the standard treatment for high-grade NMIBC (Ta, T1 and Cis), but the rate of treatment failure in these patients ranges from 20 to 40% [13]. In such cases, “early cystectomy” is recommended [4] as it results in increased 10-year cancer-specific survival (CSS). Denzinger et al. report 78% of CSS, compared to 51% when cystectomy is deferred [14]. Therefore, a quick and proper evaluation of the effectiveness of BCG therapy is pivotal in the management of high-grade NMIBC patients [15].

To obtain reliable data, BBs would seem to be imperative, but several studies questioned the need for such procedure, suggesting that the combination of less invasive procedures, namely urinary cytology and cystoscopy, could be reliable enough while avoiding another surgical procedure. Indeed, Guy et al. [16] reported that, in their cohort of 130 patients with high-risk BCa treated with BCG, BBs were not necessary when cytology and cystoscopy findings were negative. Specifically, they claimed a 100% sensitivity of such approach in detecting BCa after BCG treatment and NPV of 100%, which would allow sparing 58% of unnecessary BBs. A recent retrospective study on 21 patients affected by Cis treated with BCG pointed out a 100% NPV when cytology and cystoscopy findings were negative, again suggesting that BBs could be avoided in such patients, but a 74.1% accuracy in case of positive and/or suspicious results of one or both exams [17]. The very small cohort examined and the lack of raised lesions (Ta/T1) may, however, flaw their results. In a retrospective, single-centre study and cumulative analysis, the PPV was 63% in patients with positive cystoscopy and negative cytology and 89% in patients with positive cystoscopy and positive cytology; conversely, in patients with negative cytology and negative cystoscopy, BBs were negative in 94% of cases. Based on these findings, the authors suggested that routine BBs are not mandatory, but should rather be tailored on cystoscopy and cytology findings [6].

The present study pointed out that the diagnostic accuracy of urinary cytology was low (0.56) and was clearly outperformed by that of cystoscopy (0.70). The addition of urinary cytology to cystoscopy did not significantly improve the diagnostic accuracy (0.71) of this test. The NPV of the combination of these two tests was 90.7%. Performing BBs only in patients with positive cytology and/or positive/suspicious cystoscopy would have spared 64% (140/219) of BBs, but would have miss tumor in 9.3% (13/140) of patients, which represent approximately 30% of all BCas. On the other hand, routine BBs reduced by nearly 27% the number of patients undergoing outpatients’ cystoscopy, which is anyway a minor yet invasive procedure involving time, costs, and potential complications. These data should be taken in due account in patient counselling.

In keeping with our findings, Hara et al. [18] reported positive BBs in 15.9% of patients with negative urinary cytology and negative cystoscopy. They encouraged the use of routine BBs, since changes in mucosal appearance and cell anomalies related to denudation process following BCG treatment may affect the ability of cytology and cystoscopy in detecting BCa. Guy et al. [16] had already pointed out that bladder mucosa alterations due to BCG therapy may last up to 6 months leading to under- and over-diagnosis of recurrent BCa [16]. Indeed, BCG-related cellular anomalies may result in a high rate of false positives; in our cohort, the mean PPV of positive cytology was only 40%. Similarly, Swietek et al. [6] pointed out that the PPV of positive cytology ranges from 23 to 100% [5, 6, 18, 19]. Such data support the hypothesis that the interpretation of urine cytology findings after BCG therapy may be difficult, requiring an experienced cytopathologist, and that it may suffer from interobserver variability [20].

Another study keeping with our findings reported positive BBs in 12.8% of patients with negative urinary cytology and negative cystoscopy. Nevertheless, they suggested to avoid BBs in patients with negative exams at 3 months, but recommended BBs at 6-month follow-up irrespective of urinary cytology or cystoscopy findings [21]. Table 4 summarizes studies comparing the diagnostic performance of cytology + cystoscopy *vs.* BBs in detecting BCa after BCG induction cycle.

**Table 4:** Studies reporting the diagnostic performance of cytology + cystoscopy *vs.* BBs in detecting BCa after BCG induction cycle

	(20)Dalbagni G	(15)Guy L	(18)Skemp NM	(5)Murakami T	(17)Hara T	(16)Smith PJ	Our results
Patient, *n*	83	130	95	65	127	21	219
*Disease prevalence, % (95% CI)	30.1% (20.5–41.1)	24.6% (17.4–32.9)	21.0% (13.3–30.6)	27.6% (17.3–40.1)	21.2% (14.5–29.4)	33.3% (14.5–56.9)	19.6% (14.5–25.5)
Sensitivity, % (95% CI)	96.0% (79.6–99.9)	100.0% (89.1–100.0)	95.0% (75.1–99.8)	94.4% (72.7–99.8)	62.9% (42.3–80.6)	100% (59.0–100)	69.7% (53.8–82.8)
Specificity, % (95% CI)	12.0% (4.9–23.3)	77.5% (68.0–85.3)	53.3% (41.4–64.9)	89.3% (76.9–96.4)	53.0 (42.7–63.0)	50.0% (23.0–76.9)	72.1% (64.9–78.6)
*PPV, % (95% CI)	32.0% (29.3–34.7)	59.2% (50.1–67.7)	35.1% (29.4–41.3)	77.2% (59.5–88.6)	26.5% (20.2–34.0)	50.0% (37.2–62.8)	37.9% (31.0–45.4)
*NPV, % (95% CI)	87.5 (47.6–98.1)	100.0%	97.5% (85.4–99.6)	97.6% (86.1–99.6)	84.1% (75.8–89.9)	100%	90.7% (86.0–93.9)
*Accuracy, % (95% CI)	37.3% (26.9–48.6)	83.0% (75.5–89.0)	62.1% (51.5–71.8)	90.7% (80.9–96.5)	55.1% (46.0–63.9)	66.6% (43.0–85.4)	71.6% (65.2–77.5)
Missed BCa, *n* (%)	1 (4)	0 (0)	1 (5)	1 (5.5)	10 (37)	0 (0)	13 (30)
Avoidable BBs, *n* (%)	8 (32)	76 (58.5)	41 (43.1)	43 (66.1)	63 (49.6)	7 (33.3)	140 (63.9)

*PPV* Positive predictive value; *NPV* Negative predictive value; *BBs* Bladder biopsies; *BCa* Bladder cancer

(*) these values are dependent on disease prevalence

Questions remain whether we can improve the diagnostic ability of our diagnostic tests. As for cystoscopy, Draga et al. [22] reported that BCG treatment decreased the specificity of photodynamic diagnosis (PDD) in detecting BCa, since the false-positive rates at < 3 months and > 3 months were 59 and 44%, respectively (*p* = 0.01). BCG also reduced PDD sensitivity, which was 90% at < 3 months from last instillation, compared with standard of 97% (*p* = 0.015). Likewise, Ray et al. [23] found that the false-positive rate for PDD-guided biopsies was 63%. Having said this, it is worth mentioning that, in patients with positive cytology, the BCa detection rate of PDD was 75%, while in those with negative cytology dropped down to 14%. In other words, urinary cytology seems to reliably predict which patient might benefit from PDD after BCG treatment.

Efforts to predict response to BCG treatment also include the use of urinary and tissue molecular markers [24–27]. Unfortunately, none of them has yet entered routine clinical practice.

The main question, however, is the clinical relevance of the lesions that would be missed by omitting routine BBs. In our series, 4/13 lesions were low-grade Ta tumors. Though early (3 months) recurrence is considered a negative prognostic factor, these lesions are anyway handled by BCG maintenance [4]. Two patients had high-grade (one Ta and one T1) lesions, while 7 had Cis. Only 50% of patients with Cis 3 months after BCG are rescued by BCG re-induction, whereas the other 50% requires cystectomy with a risk of positive nodes of nearly 15% [28]. EAU guidelines also highlight that high-grade T1 disease after BCG induction is associated with a high risk of disease progression. All these information are of major relevance when counselling patients regarding the management of this challenging disease.

This study is not without limitations. First, it is a retrospective analysis even if data were prospectively collected. Second, urinary cytology was not carried out by dedicated cytologists; this might have affected the PPV of our results, since it was lower than reported in other studies. Third, not all patients underwent second TUR.

## Conclusion

To our knowledge, this is the largest study testing the value of routine BBs after BCG induction treatment. Findings suggest that performing BBs only in patients with positive cytology and suspicious/positive cystoscopy after BCG induction treatment would have spared 64% or un-necessary BBs but miss BCa in 9.3% of patients, representing almost 30% of BCas. While there are no means to determine the consequences of missing such BCas, we believe that this information is relevant in patient counselling and warrant further studies.
